# Risk Factors for Bleeding in Hospitalized at Risk Patients With an INR of 5 or More Treated With Vitamin K Antagonists

**DOI:** 10.1097/MD.0000000000002366

**Published:** 2015-12-31

**Authors:** Sophie Liabeuf, Lucie-Marie Scaltieux, Kamel Masmoudi, Bertrand Roussel, Julien Moragny, Michel Andrejak, Valérie Gras-Champel

**Affiliations:** From the Service de Pharmacologie Clinique, Centre Régional de Pharmacovigilance, Centre Hospitalier Universitaire (CHU) Amiens Sud (SL, L-MS, KM, JM, MA, VG-C); INSERM U1088, Université de Picardie Jules Verne (SL, MA, VG-C); and Laboratoire d’Hématologie, CHU Amiens Sud, Amiens, France (BR).

## Abstract

Various predictive scores for vitamin K antagonist (VKA)-related bleeding have been developed and validated in outpatients and in patients treated for specific indications (when VKAs are used under optimal therapeutic conditions). However, there are few published data on the evaluation of bleeding risk factors in hospitalized, at-risk patients (with a high international normalized ratio [INR]) treated with VKAs. The objective of the present study was to identify the most relevant bleeding risk factors in 906 VKA-treated patients with an INR of 5 or more hospitalized in a French university medical center.

Over a 2-year period, we screened all consecutive VKA-treated adults with a risk of major bleeding (defined as an INR ≥ 5 on admission). Demographic and clinical characteristics, medications, and bleeding characteristics were recorded prospectively.

The overall incidence of bleeding was 26.6% (serious bleeding: 21.4%; fatal bleeding: 5.4%). An INR ≥ 8.5, a history of recent digestive tract lesions, trauma in the preceding 2 weeks, and known noncompliance were independent risk factors for bleeding and serious bleeding.

Our present findings emphasize that VKAs should not be prescribed to patients with a high risk of bleeding (noncompliant patients and those with recent trauma or recent gastrointestinal lesions). It is essential to monitor the INR on a frequent basis and adjust oral anticoagulant treatment appropriately.

## INTRODUCTION

Despite the recent introduction of new direct oral anticoagulants (DOAs), vitamin K antagonists (VKAs) still constitute the main therapeutic option for preventing and treating thromboembolism. However, the clinical benefit of VKA treatment is counterbalanced by a thrombotic risk (related to insufficiently effective prevention) and a hemorrhagic risk (related to excessively high doses of anticoagulant).^1^

The clinical management of VKA treatment is very complex (notably due to a narrow therapeutic safety window and great inter and intraindividual variability in the response to anticoagulants) but is facilitated by monitoring the international normalized ratio (INR) and administering an antidote in the event of an overdose.^2^ In fact, the response to VKA treatment is difficult to predict; some patients with comorbidities may suffer bleeding with dramatic consequences (after even a small overdose), whereas other patients with a very high INR may not bleed at all. However, it has been suggested that in most cases, (i) a high INR is strongly associated with mortality ^3^ and (ii) an INR of 5 or more is correlated with a significant risk of major hemorrhage.^4–7^

Other risk factors for bleeding include age, gender, and co-morbidities (such as cancer, high blood pressure, diabetes, peptic ulcer disease, renal impairment, anemia, bleeding history, alcoholism, gene polymorphism, and previous stroke).^8–13^ However, the impact of a given risk factor varies from one study to another. Furthermore, other important factors (drug compliance, a patient's knowledge of his/her treatment, and use of an anticoagulation booklet, for example) have not been extensively evaluated.

Various predictive scores for VKA-related bleeding have been developed and validated in VKA-treated outpatients in general ^14^ and in patients with specific VKA indications (such as atrial fibrillation) under optimal therapeutic conditions.^12,15^ However, there are few data on the evaluation of bleeding risk factors in hospitalized, at-risk patients (those with an INR of 5 or more) being treated with VKAs. Hence, the present survey used a novel patient selection method that more closely mirrors real-life conditions; accordingly, we hypothesized that any subsequently identified bleeding risk factors could be more easily extrapolated to clinical practice.

The primary objective of the present study was to identify the most relevant bleeding risk factors in VKA-treated, hospitalized patients with an INR ≥ 5.

## PATIENTS AND METHODS

### Study Population

In this prospective study at a university medical center (Amiens, France), all consecutive VKA-treated adults presenting with a major bleeding risk (defined as an INR ≥ 5 on admission) were included over a 2-year period (from January 1, 2006 to December 31, 2007). All patients gave their consent. The study was approved by the local independent ethic committee (Comité de Protection des Personnes Nord Ouest II) and performed in accordance with the ethical principles of the Declaration of Helsinki.

The patients’ characteristics were compared according to the presence or absence of bleeding. Patients with bleeding were then divided into a minor bleeding subgroup and serious bleeding subgroup.

### Data Collection

Patients were prospectively selected on the basis of the INR measured in the university medical center's hematology laboratory. All patients with INR ≥ 5 were included in the study if they had been treated with VKAs before or during hospitalization. Each patient could be included only once.

For each patient, the following characteristics were recorded prospectively after questioning the physician, the medical staff, and the patient and by consulting the patient's hospital records:

1. Demographic characteristics (age and gender) and medical history (including treated hypertension, diabetes, hypercholesterolemia, cancer, gastrointestinal lesions in the preceding 3 months, chronic kidney disease, alcoholism, surgery in the preceding 3 months, stroke in the preceding 3 months, trauma in the preceding 2 weeks, and infection in the preceding 2 weeks; for alcoholism, only the patient's physician and medical records were consulted).
2. Characteristics of the treatments (name of the drug, dose, treatment start date, indication for VKAs, any associated drugs, person administering the treatment, data on treatment compliance, regular use of an anticoagulation booklet, patient education (the patient was asked if he/she could remember being given an explanation about treatment with VKAs), recent changes in the VKA dosage and/or regimen, and INR values (measured on admission, in any previous laboratory tests and during hospitalization). Information on the INR before hospitalization was gathered using a variety of methods until a value was obtained. First, patients were questioned about their latest laboratory results. If these data were not available, the patient's hospital records were analyzed. Lastly, the patient's general practitioner was contacted, if necessary.
3. Treatment outside the scope of the current French guidelines was defined as
  - off-label prescription (ie outside the indications given in the French summary of product characteristics [SmPC])
  - inappropriate treatment with regard to a previous INR value (ie the lack of VKA dose adjustment in a patient with an INR outside the therapeutic range)
  - previous noncompliance known to medical staff. Appropriate medical care associated with next INR was defined as a VKA dose adjustment according INR target (decrease or withdrawal).
4. Characteristics of the bleeding (type, site, date of onset, severity, treatment, and outcome).

All data were entered into a computer database (Access 2003, Microsoft Corporation, Redmond, WA).

### Measured Variables

Calculation of the INR: the prothrombin time was expressed as a percentage of activity (the prothrombin rate) after measurement with an automated device (STA R, Diagnostica Stago). The thromboplastin used was Neoplastin CI (Diagnostica Stago), which has an international sensitivity index of ∼1.7.

Severe renal impairment was defined as a creatinine clearance rate (calculated using the simplified Modification of Diet in Renal Disease equation) ≤30 mL/min.

Bleeding was classified as major or minor by applying the explicit criteria defined by the International Society on Thrombosis and Haemostasis.^16^ Major bleedings were those that result in death, are life-threatening, cause chronic sequelae, or consume major health-care resources.

### Data Analysis

Data were expressed as the mean ± SD (range), the median, or the number (frequency), as appropriate. The study participants were stratified according to the presence or absence of events. Intergroup comparisons were performed with a chi squared test (for categorical variables), Student's *t* test, or a Wilcoxon–Mann–Whitney test (for continuous variables).

Univariate logistic regression analyses were used to select factors that were associated with the bleeding risk. Lastly, multivariate logistic regression analyses were performed for the bleeding risk in general, and for the serious bleeding risk) by including explanatory variables that were found to be significantly associated with bleeding in the univariate analyses. If 2 variables were correlated, only 1 was included in the model. The “age” variable was always added to the model.

Statistical analysis was performed with SPSS software (version 18.0, SPSS Inc, Chicago, IL) for Windows (Microsoft Corporation). In all tests, the threshold for statistical significance was set to *P* < 0.05.

## RESULTS

Over the 2-year inclusion period, 906 hospitalized patients were included in the study. All were receiving VKAs and had an INR ≥ 5. Overall, 241 patients (26.6%) experienced clinically apparent bleeding and 665 did not. In ∼30% of cases, hospitalization had been prompted by a family physician's observation of an excessive INR. This percentage was 52% in the bleeding group.

### Characteristics of the Bleeding

Eighty percent of the 241 patients with bleeding were classified as having a serious ADR. Table 1 shows the types of the bleeding as a function of their severity. Of the 194 patients with serious ADRs, 17% (n = 33) had a life-threatening ADR. This led to immediate death in 17 cases (9%). However, the outcome was favorable in the great majority of cases. Bleeding complications affected a variety of organs, although some sites were immediately life-threatening (intracranial, pericardial, intra-abdominal, and digestive hemorrhages, associated respectively with 60%, 45%, 40%, and 20% of the deaths). A given patient could have bleeding at >1 site.

**TABLE 1:**
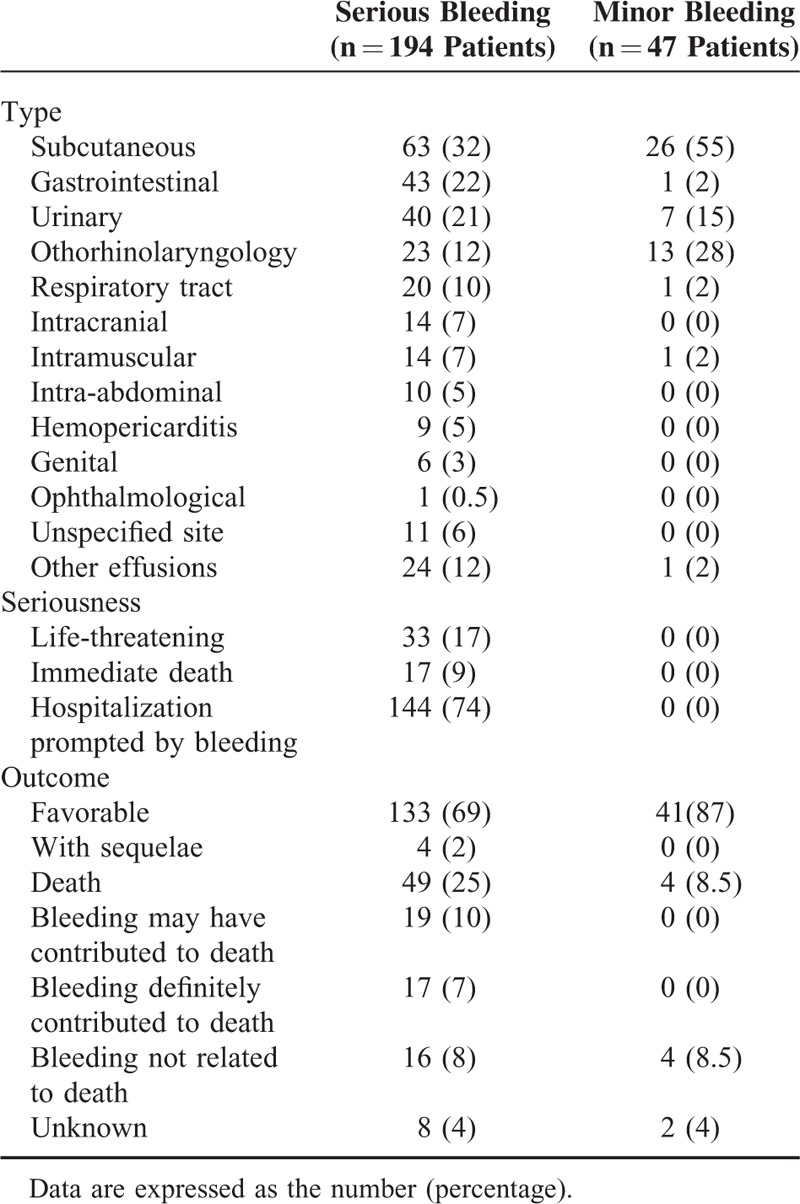
Distribution of the Bleeding in Patients, by Seriousness

### Characteristics of the Patients (Table 2)

The mean ± SD (range) age of the study population was 76.6 ± 12.4 (19–100), with a median of 77. Four hundred and thirty-nine of the patients (48.5%) were men. Gastrointestinal lesions in the preceding 3 months and trauma in the preceding 2 weeks were significantly more frequent in the bleeding group than in the nonbleeding group (<0.0001). Alcoholism was reported significantly more frequently in patients with bleeding than in patients with no bleeding. There were no other statistically significant intergroup differences in terms of the demographic or medical characteristics.

**TABLE 2:**
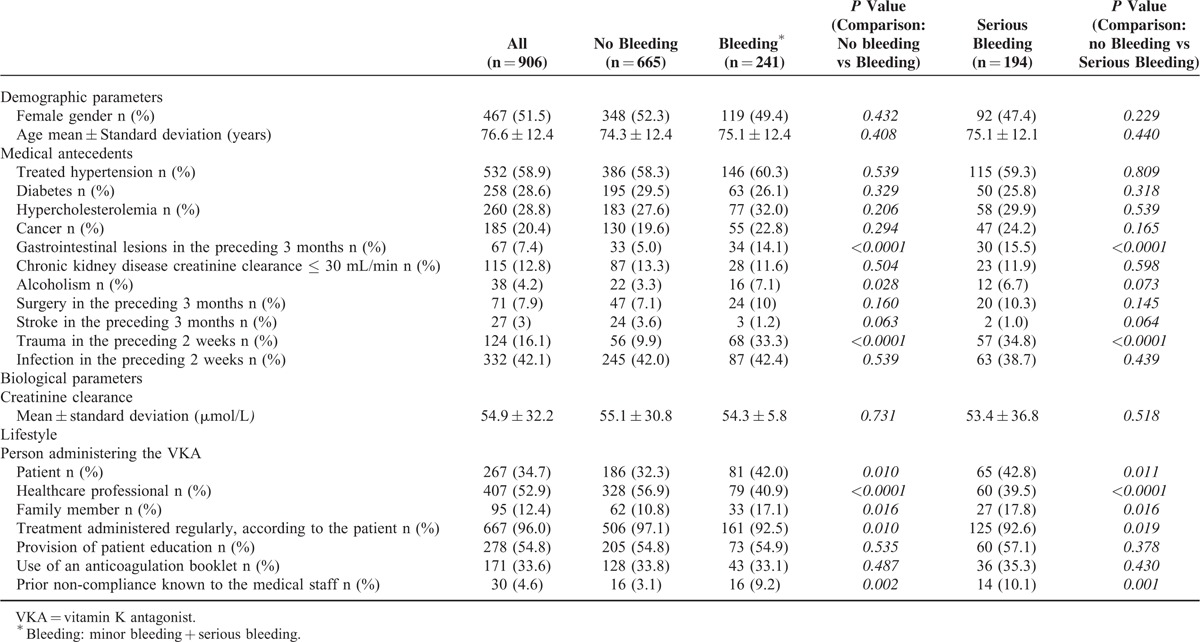
Demographic, Clinical, and Biochemical Characteristics of the Study Population

### Characteristics of the VKA Treatment (Tables 2 and 3)

The most frequent indications for treatment with VKAs were atrial fibrillation (n = 548), the presence of a mechanical or biologic replacement heart valve (n = 101), peripheral deep-vein thrombosis (n = 267), and pulmonary embolism (n = 177). The mean duration of VKA therapy was 3.5 ± 6.2 years (median: 0.6 years), with a duration <3 months in 37.2% of cases, between 3 and 12 months in 7% and >12 months in 50%. On the day of admission, the mean INR was 7.4 ± 3.4 and the median INR was 6.1. In the groups of patients with bleeding and serious bleeding, the mean values were significantly higher (8.5 ± 4.2 and 8.7 ± 4.4, respectively), when compared with the nonbleeding group (*P* ≤ 0.001 for both comparisons). A third of the patients with bleeding had an INR ≥ 8.5. We were able to obtain prehospitalization INR values for 620 of the 906 included patients; 42% of these values were within the therapeutic range. Patients with bleeding were less likely to have an INR within the therapeutic range.

**TABLE 3:**
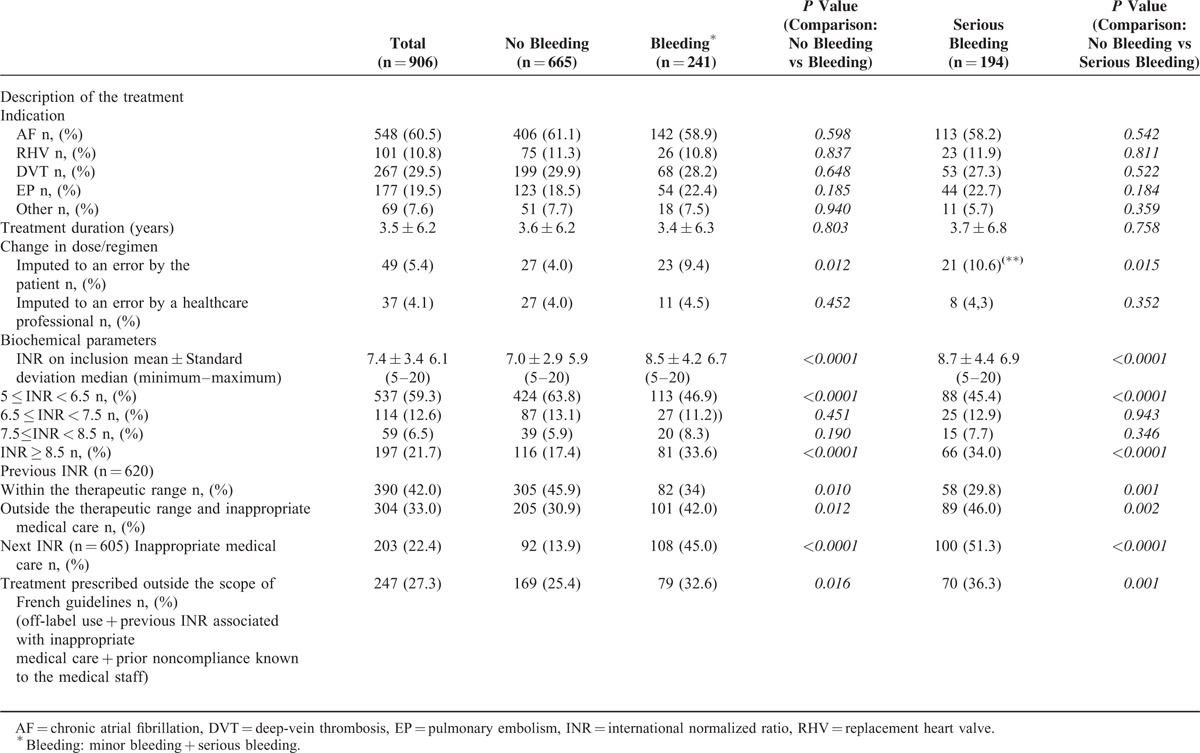
Characteristics of the VKA Treatment

The INR values measured during hospitalization were inappropriately managed in 45% of the patients with bleeding, 51.3% of the patients with serious bleeding, and 13.9% of patients with no bleeding. In 247 cases (27%), the prescription had not complied with the treatment's SmPC (ie off-label indications, an excessively high previous INR with inappropriate care, and previous noncompliance observed or strongly suspected by healthcare staff). This was true for 32.6% of the patients in the bleeding group, 36.3% of the patients in the serious bleeding group, and 25.4% of the patients in the nonbleeding group.

It appeared that VKA self-administration or VKA administration by a family member was associated with an elevated risk of bleeding. Conversely, the administration of treatment by a healthcare professional was associated with a lower risk of bleeding (Table 4). Patient education and the use of anticoagulation booklet did not appear to influence the likelihood of bleeding. Lastly, noncompliance was significantly more frequent in the 2 bleeding groups than in the nonbleeding groups (*P* < 0.001).

**TABLE 4:**
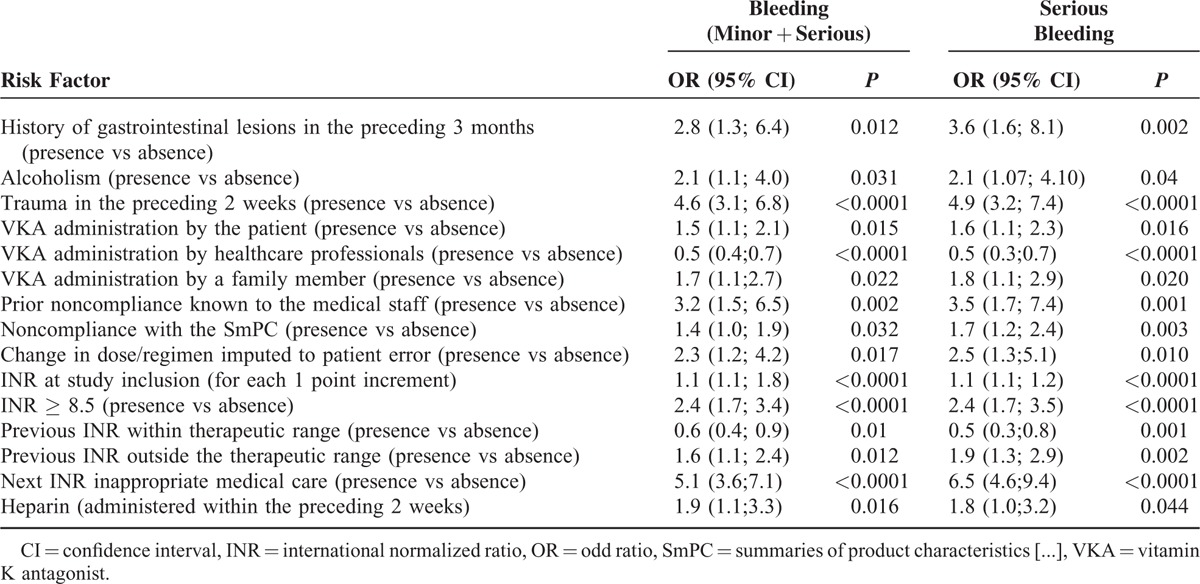
The Results of the Univariate Logistic Regression Analysis

### Drugs Combined With VKAs

On inclusion in the study, the patient was taking an average of 7.0 ± 3.3 drugs (1–23) at the same time as VKAs. In 79% of cases, at least 1 associated drug was known to interact with VKAs (mean: 1.6 ± 1.2 [1–7]). A total of 82% of the coprescriptions featured potential interactions and 73% of the coprescriptions were for long-term regimens. There were 2 absolutely contraindicated drug combinations (high-dose aspirin in 1 case, and miconazole in the other), 21 discouraged combinations (nonsteroidal anti-inflammatory drugs (NSAIDs) in 12 cases, moderate-dose aspirin in 8 cases and 5-fluorouracil (5FU) in 1 case), and 1395 drug combinations necessitating caution. The most frequently associated drugs were statins (27.8%), amiodarone (24.6%), antibiotics (20.6%), acetaminophen (17.8%), platelet aggregation inhibitors (12%), thyroid hormones (10%), corticoids (9.5%), heparin (8.8%), specific serotonin reuptake inhibitors (7.9%), and allopurinol (7%). The drugs combined with VKAs during the 2 weeks before inclusion were mainly antibiotics (primarily fluoroquinolones) (19.4%), heparin (7.1%), acetaminophen (5.3%), and platelet aggregation inhibitors (primarily aspirin) (5.3%). Only the combination of recent heparin treatment with VKAs appeared to be associated with an elevated risk of bleeding (10.5% of cases in the bleeding group vs 5.7% in the nonbleeding group; *P *≤ 0.05).

### Risk Factors Influencing Bleeding and Serious Bleeding

In a univariate analysis, we identified a number of variables associated with bleeding (whether minor or serious) and serious bleeding (Table 4). In a multivariate analysis, an INR ≥8.5, a history of recent gastrointestinal lesions, and a history of recent trauma and prior noncompliance known to the medical staff were identified as independent risk factors for bleeding and serious bleeding (Tables 5 and 6).

**TABLE 5:**
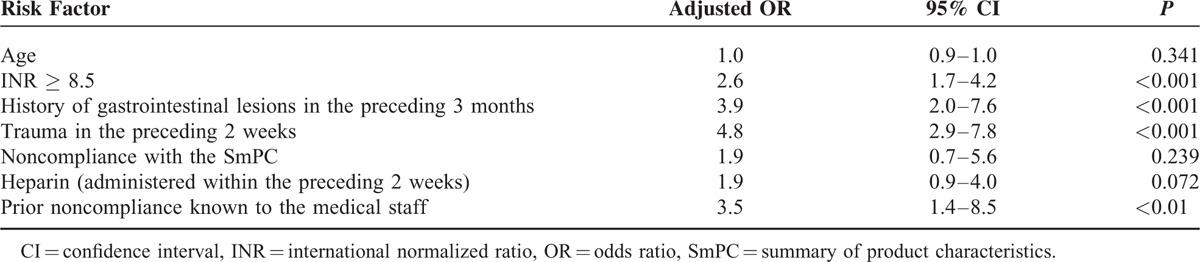
Multivariate Logistic Regression Analysis of Risk Factors for Bleeding (Minor + Serious)

**TABLE 6:**
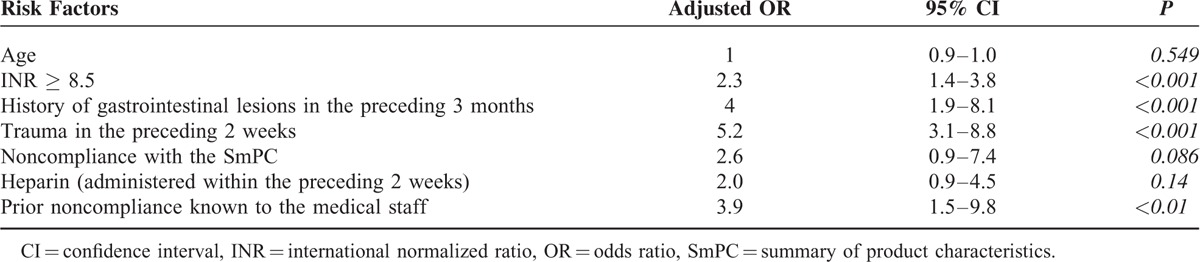
Multivariate Logistic Regression Analysis of Risk Factors for Serious Bleeding

## DISCUSSION

The incidence of bleeding in 906 hospitalized, high-INR, VKA-treated patients was 26.6% (21.4% if only serious bleeding was taken into account and 5.4% if fatal bleeding was taken into account). The incidence of serious bleeding recorded here is markedly higher than the values reported in randomized clinical trials (usually <2%). However, our study was performed in patients at a high risk of bleeding (INR ≥ 5), and it is well known that clinical trials underestimate the “real-life” incidence of adverse events. Indeed, Levi et al found that 40% of VKA users admitted to hospital for bleeding do not fulfill the eligibility criteria for the pivotal clinical trials of this type of drug.^6^ Hence, the risk of bleeding rose sharply with the number of exclusion criteria fulfilled (up to 15-fold for subjects meeting >2 exclusion criteria). These exclusion criteria would have applied to many of the participants in our study: noncompliance, alcohol dependency, concomitant medication with another antiplatelet agent, an elevated likelihood of bleeding, stroke, and cardiac comorbidities.^6^

Although bleeding episodes potentially concern all organs, some sites are associated with immediately life-threatening or functional complications. Those affecting the central nervous system are clearly among the most severe.^17,18^ In the present study, 14 patients presented with intracranial hemorrhage. Eight of these patients died and 2 others had sequelae.

Out of a wide range of potential risk factors assessed in the present study, we found that an INR ≥ 8.5, a history of recent digestive lesions, and a history of recent trauma and prior noncompliance known to the medical staff were independent risk factors for bleeding in general and serious bleeding in particular.

### Age

In our study and in the various literature reports, the mean age was high (50% of our patients were over the age of 75). However, this is partly due to the greater likelihood of indications for VKAs in elderly patients. The question of whether the frequency of VKA-related bleeding is indeed higher in older patients is subject to debate. Some researchers have reported that age >75 is associated with an elevated risk of bleeding, and so have included this parameter in the calculation of bleeding risk scores.^14,15^ In contrast, other studies (including the present study) have not confirmed this association.^19–21^ Nevertheless, there is a consensus that the increased risk of bleeding in the elderly is clearly a multifactorial phenomenon (poor drug elimination, a greater likelihood of drug interactions due to the higher number of concomitant medications).^14,22^

### Comorbidity

High blood pressure, cerebrovascular disease, recent surgery or trauma, neoplasia, heart disease, diabetes mellitus, kidney failure, liver failure, a history of gastrointestinal bleeding, and chronic alcoholism have all been described as risk factors for bleeding.^8,12,23^ Some factors are more specifically linked to certain bleeding sites: for example, hypertension, cerebrovascular disease, and head trauma are risk factors for intracranial hemorrhage.^18,24^ This association was also observed in the present study. Recent trauma emerged as an independent risk factor for bleeding of any type but was a major risk factor for intracranial hemorrhage. We also confirmed the clearly elevated gastrointestinal bleeding risk during VKA therapy in the presence of digestive mucosal alterations (polyps, ulcers, esophageal varices, etc).

Several literature studies have consistently reported an elevated risk of bleeding in patients with renal failure (even when the latter is only moderate).^23,25,26^ In the present study, a history of severe renal failure or poor renal function at baseline was not found to be a risk factor for bleeding. We also observed that alcoholism was associated with bleeding. This association has not always been found in the literature; this disparity may reflect a failure to diagnose and record alcoholism in other studies, rather than the absence of an association.^27^

### Treatment Compliance

Patient compliance with VKA treatment appears to have a key role in maintaining the INR within the therapeutic range; indeed, noncompliance has been reported as a cause of INR instability.^28^ Of all the factors tested by Davis et al, compliance was the key determinant in satisfactory anticoagulation control.^29^ In the present study, 9.2% of the patients with bleeding were known to be noncompliant at the time when their INR ≥ 5 was recorded.

In this context, patient education appears to be an essential means of encouraging patients to comply with the prescribed treatment.^30^ In the present study, patient education was not significantly associated with the risk of bleeding. However, our study (with a single question on whether information on VKA treatment was provided or not) was not designed to distinguish between mere information and “true” patient education (ie repetition of information, with regular monitoring of knowledge retention). Furthermore, our study was not designed to assess the patient's knowledge of his/her treatment; we can merely affirm that a marked percentage of patients (43–45%, depending on the group) did not remember receiving any information about their VKA treatment.

#### Drug Interactions

Many drugs are known to interact with VKAs, with the level of risk ranging from an absolute contraindication to a simple precaution. In the present study, a median of 7 drugs were combined with the VKA. About 2.4% of the combinations were discouraged (mainly moderate-dose aspirin, NSAIDs, and 5FU) and 2 combinations were absolutely contraindicated (high-dose aspirin and miconazole). Of the 17,861 patients studied by Snaith et al,^31^ 68% of the coprescriptions included at least 1 drug that can interact with warfarin, and most coprescriptions were taken for long periods (59%). Here, we found similar proportions: 82% of the co-prescriptions featured potentially interacting drugs and 73% of the coprescriptions were for long-term regimens. Only heparin emerged as a risk factor for bleeding in the univariate analysis; these were mostly situations in which heparin was not withdrawn when the INR came back into the therapeutic range. Although most recently introduced drugs have not been identified as risk factors for immediate bleeding, the most frequently recorded drugs (antibiotics, acetaminophen, and antiplatelet agents) are well-documented potentiators of VKAs.^31^ These observations raise the question of whether these drugs contribute to an abnormally high INR during VKA treatment.

### INR

Regardless of the indication, most studies show that the intensity of anticoagulation therapy is a risk factor for bleeding—with a significantly elevated incidence when the INR exceeds a value of 4 or 5.^15,17,18,23^ In a study of 42,451 patients, Oden et al showed that mortality is closely correlated with the INR; the risk per unit doubled when the INR exceeded 2.5.^3^ Consistently, we found that the mean INR was significantly higher in the bleeding group than in the nonbleeding group (*P* ≤ 0.001). In an adjusted analysis, the risk of bleeding rose by a factor of 2 when the patient's INR was ≥8.5.

An unstable INR might be a risk factor for bleeding. In the literature, the criteria used to assess instability include (i) the time spent within the therapeutic range, (ii) the percentage of INR values within the therapeutic range, (iii) the INR's SD or variance, and (iv) the number of dose adjustments required. Our study was not designed to assess these criteria. In fact, we focused on the preceding INR value. This value was already abnormally high (with regard to the specifications in the SmPC) in >40% of the patients in the bleeding group and was significantly higher in the latter than in the nonbleeding group (*P* ≤ 0.01). It is likely that prescribers underestimated the importance of the INR in adaptation of the VKA dose (as 21.7% of the patients had an INR ≥ 8.5). In many papers dealing with VKAs, INR monitoring is indeed described but is “diluted” by other information and is therefore not perceived to be one of the foremost hemorrhagic risk factors. It is clear that closer INR monitoring can minimize the risk of bleeding.^32^

The “ideal anticoagulant” would be associated with high safety (ie a low incidence of ADRs in general and hemorrhage in particular), no interindividual variability, no food or drug interactions, without biological control and the ready availability of a specific antidote. The continuing quest for an “ideal anticoagulant” led to the recent approval of the DOAs dabigatran, rivaroxaban, and apixaban. The US Food and Drug Administration (FDA) compared bleeding rates for dabigatran and warfarin by analyzing insurance-claim data and administrative data from its Mini-Sentinel database. The FDA found that bleeding rates associated with dabigatran use during the period of interest did not appear to be higher than those associated with warfarin.^33^ However, management of the bleeding risk with these new drugs is still sketchy, due to the lack of a specific antidote and the absence of anticoagulation monitoring.^34^ This is an important limitation, as the results of the present study demonstrated that anticoagulation monitoring is one of the most important determinants of the risk of bleeding. As DOAs have only recently gained marketing approval, clinicians do not yet have extensive experience with these drugs—especially in patients at a high risk of bleeding. Hence, the decision to extend the use of DOAs will depend on weighing up the risk of major bleeding against the risk of thromboembolic events. It is noteworthy that with the exception of the INR, the other risk factors for bleeding identified in the present study are not specific to VKAs and thus might be transposable to DOAs.

The limitations of the present study include the lack of a pharmacogenetic evaluation (which might explain the variability in the INR); this may have prevented us from taking account of genetic risk factors. A second limitation relates to the lack of precision in establishing whether or not patient education was provided; a single question was asked, and the answers may have suffered from memory bias. A third limitation was due to the INR-based selection criterion: we therefore had to focus on VKAs and could not study DOAs. Hence, it might not be possible to extrapolate our present results to all oral anticoagulants. The present study's main strengths are the large sample size, the exhaustiveness of the inclusion process (as we included all VKA-treated patients with an INR ≥ 5 or more admitted to our university medical center over a 2-year period) and, lastly, the large number of bleeding risk factors surveyed.

In conclusion, we found that well-known risk factors are independently associated with a risk of serious bleeding in hospitalized, high-INR patients. Our present findings emphasize that VKAs should not be prescribed to patients with a particular risk of bleeding (noncompliant patients and those with recent trauma or recent gastrointestinal lesions). It is essential to monitor the INR regularly and manage it appropriately during oral anticoagulant treatment.
